# Supplementary material for: Learning curve and surgical outcome of robotic assisted colorectal surgery with ERAS program
Source: Sci Rep. 2022 Nov 29;12:20566. doi: 10.1038/s41598-022-24665-w (PMC9709162; doi:10.1038/s41598-022-24665-w)
Supplement: Supplementary file 2 — Supplementary Information 2. [file 41598_2022_24665_MOESM2_ESM.zip › RAL Raw data-Ñ[▒K/100/OP N_ñw╝╨░O▒Kñσ.pdf]

W83 / W83

臺中榮民總醫院
手術室護理記錄

[redacted]

基本資料

- 室別： 開刀房-17室，手術科別： CRS，手術類別： 常規，日期： 2021/07/22
- 進入時間： 1400 ； 麻醉開始時間： 1405 ； Time Out時間： 1508 ； 劃刀時間： 1510 ； 手術結束時間： 1845 送出時間： 1905 ；
- 麻醉方式： GE
  診斷：RECTAL CA, U/3 WITH LOCAL ADVANCED FOR POSSIBLE ROBOTIC
  手術名稱：Robot Assisted Colectomy(L)
  手術局部用藥：NIL
  術前預防性抗生素：
    - 抗生素名稱一： Veterin 1GM IVD/由麻醉人員執行
    - 時間： 1500
    - 上次簽章: 林雨璇
  ，追加預防性抗生素：
- 手術醫師： 蔣鋒帆，楊曙榮，陳映晴
- 麻醉醫師： VS 齊光彪
-
    - 刷手護士A ： 高子貽，時間： 1400，刷手護士B ： 蘇敬涵，時間： 1400，接替刷手一： 歐真勤/蘇敬涵，時間： 1500，接替刷手二： 吳貞儀，時間： 1800
    - 流動護士A ： 林雨璇，時間： 1400，接替流動一： 高子貽，時間： 1500，接替流動二： 林裕瑋，時間： 1800
- 更多接替及流動護士： 接替流動三： 林怡廷，時間： 1900

皮膚狀況

A-V Shunt： 無
術前： 完整
記錄者： 上次簽章: 林雨璇
術後： 完整
記錄者： 林怡廷

手術準備

手術臥式 單 手術臥式

-
    - 手術臥式： 垂頭仰臥
    - 使用附件： 溫毯 38 ℃，頭圈，腳架，其它： 輔助海棉墊
    - 皮膚準備： 10%Aq Betadine，Hibiscrub，2% Chlorhexidine
    - 電燒： 使用電燒: 電極片位置： 其它： 電燒迴路墊，雙極電燒，記錄者： 上次簽章: 林雨璇

止血帶T： 無

植入物

- 植入物名稱/號碼/數量/部位：da Vinci Xi EndoWrist Stapler 45 Green Reload-48445G*2釘/腸吻合處
  coviden EEA 29mm/腸吻合處

，記錄者： 上次簽章: 林裕瑋

標本送檢

- 細菌培養 無
- 細胞學 無
- 冰凍切片 無
- 病理標本
  組織名稱一：rectum+R't ovary
  ，
  組織名稱二：IMA root
  ，

組織名稱三： DISTAL CUT END
，更多組織名稱
  - 組織名稱四： peritioum
- 截肢 無

標本交付 標本： 3 袋 ， 標本： 0 盒 ， 標本： 1 瓶 ， 細胞學： 0 管 ， Culture Tube： 0 管 ， 被交付醫師： R 陳映晴，

- 記錄者： 上次簽章: 林裕瑋
- 時間： 1850

傷口術後數料

傷口數料： 傷口數料： 無

- 請於圖上標示部位：

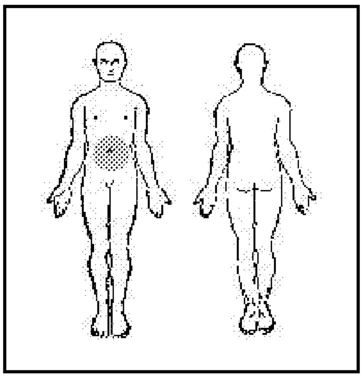

- 傷口數料： mepore

導管

導管	天數	導管資訊	插入日期	插入時間	更換日期	拔除日期	拔除時間	拔除原因	預計拔除/更換	預計拔除/更換日期
Epidural PCA(1)	1	部位:硬脊膜;	2021/07/22	1410						
Foley(1)	1	號碼:16 Fr.; 部位:膀胱;	2021/07/22	1900						
Double Lumen CVC(1)	1	號碼:8 Fr.; 深度:15cm; 部位:jugular (右); 象限:RU;	2021/07/22	1430						
A-line (1)	1	號碼:20 G; 部位:左手;	2021/07/22	1420						

用物計數

- 手術中出血量： minimal c.c.
- 手術中尿量： 300 c.c.
-
  - 其它： 無

用物計數： 多切口同時縫合

- 1.器械： 正確，2.縫針： 正確，3.刀片： 正確，4.紗布： 正確，5.腹部墊： 未使用，6.紗球： 未使用，7.PEANUTS ： 未使用，8.紗條： 未使用，9.絲帶： 未使用，10.針頭： 未使用，11.Vessel loop： 未使用，12.Boots： 未使用，13.Jelco： 未使用，14.棉條： 未使用，
  - 第一次：
    - 刷手護士 ： 歐真勤
    - 流動護士： 上次簽章: 高子貽
    - 時間： 1745

  ,
  - 第二次：
    - 刷手護士 ： 歐真勤
    - 流動護士： 上次簽章: 高子貽
    - 時間： 1753

  ,
  - 第三次：
    - 刷手護士 ： 吳貞儀

- 流動護士： 上次簽章: 林裕瑋
- 時間： 1815

備註

備註： Davinci XI 30度 Scopy No.1

- 記錄者： 上次簽章: 林雨璇
- 時間： 1500

填寫護理人員 林怡廷

正式摘要

		W83 / W83			

doctorRank:B
